# Dietary intake of genistein suppresses hepatocellular carcinoma through AMPK-mediated apoptosis and anti-inflammation

**DOI:** 10.1186/s12885-018-5222-8

**Published:** 2019-01-03

**Authors:** Sang R. Lee, Sun Woo Kwon, Young Ho Lee, Pelin Kaya, Jong Min Kim, Changhwan Ahn, Eui-Man Jung, Geun-Shik Lee, Beum-Soo An, Eui-Bae Jeung, Bae-keun Park, Eui-Ju Hong

**Affiliations:** 1 College of Veterinary Medicine, Chungnam National University, 99 Daehak-ro, Suite 401Veterinary medicine Bldg., Yuseong, Daejeon, 34134 South Korea; 2 Translational Xenotransplantation Research Center, Seoul National University, Seoul, Republic of Korea; 3 College of Veterinary Medicine, Chungbuk National University, Cheongju, Chungbuk Republic of Korea; 4 Kangwon National University, Chuncheon, Gangwon Republic of Korea; 5 Department of Biomaterials Science, College of Natural Resources & Life Science, Pusan National University, Miryang, Republic of Korea

**Keywords:** HCC, Genistein, AMPK, Inflammation, Apoptosis, Phytoestrogen

## Abstract

**Background:**

Women have a lower risk of hepatocellular carcinoma (HCC) than men, and the decreased possibility of HCC in women is thought to depend on estrogen levels. As a soybean-isoflavone product, genistein has estrogenic activity in various reproductive tissues, because it mimics 17β-estradiol and binds the estrogen receptor. Though genistein is a known liver cancer suppressor, its effects have not been studies in long-term experiment, where genistein is fed to a female animal model of HCC.

**Methods:**

Mice were treated with diethylnitrosamine (DEN) to induce HCC at 2 weeks of age and fed with supplemental genistein for 5 months, from 40 to 62 weeks of age.

**Results:**

The dietary intake of genistein decreased the incidence of HCC and suppressed HCC development. Genistein induced phospho-AMPK in total liver extracts, Hep3B cells, and Raw 264.7 cells, and phospho-AMPK promoted apoptosis in liver and Hep3B cells. Moreover, phospho-AMPK down-regulated pro-inflammatory responses and ameliorated liver damage. A suppressed pro-inflammatory response with increased mitochondrial respiration was concomitantly observed after genistein treatment.

**Conclusions:**

Genistein-mediated AMPK activation increases hepatocyte apoptosis through energy-dependent caspase pathways, suppresses the inflammatory response in resident liver macrophages by increased cellular respiration, and consequently inhibits the initiation and progression of HCC.

**Electronic supplementary material:**

The online version of this article (10.1186/s12885-018-5222-8) contains supplementary material, which is available to authorized users.

## Background

Women have a lower risk of hepatocellular carcinoma (HCC) than men, and this difference of HCC risk is most pronounced in individuals who are under 50 years of age [1]. The decreased incidences of HCC in women is thought to depend on sex hormone levels, since estrogen suppresses pro-inflammatory cytokine and hepatocyte growth factor levels during liver injury and HCC [2, 3]. Estrogen has been discovered to play a major role in HCC retardation, and researchers have focused on the effects and mechanism of action of estrogen as an HCC therapy [4].

As a soybean-isoflavone product, genistein has estrogenic activity in various reproductive tissues because it mimics 17β-estradiol and binds the estrogen receptor [5]. Interestingly, this phytoestrogen is also known to suppress the growth of gastric and liver cancer cells by triggering apoptosis [6]. Moreover, a recent study found that genistein promotes HCC cell death through suppressing glycolysis. Among various ingestible dietary phytoestrogens, genistein most effectively reduced HCC risk in patients [7]. However, most experiments have focused on therapeutic effects in vitro. Limited in vivo experiments have been performed with genistein, and few studies have included clinical applications [6].

Genistein activates AMP-activated protein kinase (AMPK) and subsequently suppresses nuclear factor kappa B (NF-κB) signaling [8, 9]. In hepatocytes, AMPK activation is necessary for energy metabolism, ATP production, and mitochondrial biogenesis [10]. After its activation by phosphorylation, phospho-AMPK suppresses the growth of HCC cells [11]. In an early study, the level of AMPK expression was found to inversely correlate with HCC risk [12]. Therefore, AMPK has great potential as a therapeutic target, for HCC prevention and treatment [13]. AMPK activation induces apoptosis through the p53 pathway, which exhibits anti-cancer activity and negatively correlates with NF-κB activation [14–17]. AMPK also contributes to oxidative phosphorylation, which is a major metabolic pathway of normal cells for energy production [18]. Interestingly, genistein weakens the pro-inflammatory response by down-regulating inflammation, via AMPK activation and subsequent NF-κB suppression [19]. When genistein induces apoptosis in various types of cancer cells [6], genistein is expected to suppress HCC by anti-inflammatory and pro-apoptotic pathways; however, its long-term effects have not yet been investigated.

To avoid changeable female-dependent factors, many research groups have investigated HCC development using male rodent models. However, recent studies have suggested that inclusion of both sexes is necessary to identify potential molecular mechanisms. In present study, we used a female mouse model after natural cessation of menstrual cycling (menopause), and female mice were fed with supplemental genistein for 5 months, from 40 to 62 weeks. This study was necessary because no long-term experiments of genistein-fed female animals bearing HCC have been performed. In addition, it will be useful to compare the results obtained here to the studies done on male rodent model. In this study, we focused on the anti-tumor effect of genistein and investigated key mediators under female condition.

## Methods

### Animals and treatment

Mice on a C57BL/6 N background were obtained from Orient Bio (Daejeon, Korea) and housed in a pathogen-free facility at Chungnam National University under a standard 12 h light:12 h dark cycle and fed standard chow with water provided ad libitum. All mouse experiments were approved and performed in accordance with the Chungnam Facility Animal Care Committee. To develop HCC, 2-w-old female mice were administered diethylnitrosamine (DEN, 25 mg/kg body weight; 73,861; Sigma) in a 0.9% saline solution by i.p. injection, as shown in previous report [20]. Genistein was fed (80 mg/kg/day, 2 mg/day) with chow for 5 months from 40 weeks to 62 weeks of their age. The animals were monitored thereafter by palpation for tumor formation and killed by cervical dislocation at the indicated time points for serum and liver isolation. Mice used for experiment were at least 6 for each control and genistein group.

For acute DEN experiments, mice were administered DEN (100 mg/kg body weight) in a 0.9% saline solution by i.p. injection, and livers were isolated at the indicated time points following injection. Ovariectomy was performed 2 weeks before DEN treatment, by incision on the dorsal midline to reduce pain than ventral side. Mice were administered genistein in a corn oil by subcutaneously (200 mg/kg body weight), each half for morning and night with 12 h interval and 1 day before DEN injection. Mice used for experiment were at least 3 for each group.

### Cell culture

All cell culture reagents were purchased from Welgene (Gyungsan, Korea). Hep3B human hepatocyte cancer cell and Raw 264.7 mouse kupffer cell were maintained at 37 °C in a 5% CO2 atmosphere in DMEM (Welgene, LM001–05) supplemented with 5% (vol/vol) fetal bovine serum, penicillin (100 U/mol) and streptomycin (100 μg/ml). For experiments, cells were maintained in a steroid-free condition with phenol free DMEM (Welgene, LM002–05) media containing 2% charcoal dextran treated-FBS, penicillin (100 U/mol) and streptomycin (100 μg/ml). Genistein (LC laboratories, G-6055) and cobalt(II) chloride hexahydrate (Sigma, C8661) were added every 12 h with media change. Lipopolysaccharides (LPS, Sigma, L6511) was added after dissolved in distilled water (100 ng/ml). All cell experiments were repeated at least 3 times.

### Western blotting

Cell and liver samples were homogenized in lysis buffer (10 mM Tris, pH 7.5, 150 mM NaCl, 1% Triton X-100, 1 mM phenylmethylsulfonyl fluoride, 0.2 mM sodium orthovanadate, 0.5% Nonidet P-40 containing protease inhibitor phenylmethylsulfonyl fluoride (P7626; Sigma-aldrich, St. Louis, MO, USA) at 4 °C for 15 mins. TissueLyser II (Qiagen, Hilden, Germany) and sonicator (Qsonica, Newtown, CT, USA) were used for protein lysis. Protein was quantified using the Bradford assay (PRO-MEASURE, Intron Biotechnology, sungnam, Korea) at 595 nm and proteins were resolved on 5–12% SDS-PAGE gels (Running buffer: 25 mM Tris, 192 mM Glycine, 0.1% SDS, and D.W.). After electrophoresis, gels were blotted with a PVDF membrane (IPVH 00010, millipore) at 350 mA for 1–2 h with transfer buffer (25 mM Tris, 192 mM Glycine, 20% (*v*/v) Methanol, and 3rd D.W.). The membranes were then blocked for 1 h in PBS containing 0.1% Tween 20 (PBS-T) and 5% skim milk, and then incubated in tube rotator for overnight at 4 °C with primary antibodies (see below) in the 3% BSA buffer (1:1000–1:2500). Membranes were then washed three times in PBS-T for 30 mins to remove excess antibody and then incubated for overnight at 4 °C with secondary antibodies in PBS-T + 5% skim milk (1:5000–1:10000): mouse anti-rabbit (bs-0295G-HRP; MA, USA or 211–032-171; Jackson laboratories Inc., PA, USA) or goat anti-mouse (115–035-174; Jackson laboratories Inc., PA, USA). Following 3 washes in PBS-T, immunoreactive proteins were detected by using Ultra 2.0 Western Blotting substrate (XLS075,1000, Cyanagen) ECL solution. All buffers used for phospho-protein were based on TBS and BSA, rather than PBS and Skim milk.

Primary antibodies used were: Cleaved Cas-3, Cas-3 (CST, #9930 T), PARP (CST, #9532), Actin (sc-1616, Santa cruz), P-AMPK, AMPK (CST, #9957), and p53 (Abnova, PAB12719).

### Histological and Immunohistological analyses

For histology, livers were fixed in 10% neutral buffered formalin and processed through serial dehydration steps. Tissues in paraffin blocks were sectioned by microtome (RM2135, Leica) at 4 μm. Sections were attached to silane coated slides (5116-20F-C, Muto), stained with Hematoxylin and Eosin, and examined by light microscopy. For immunohistochemistry, slides were antigen retrieved with 0.1% sodium citrate buffer (CA2081, Georgiachem) at 95 °C for 60 mins. After blocking with 3% bovine serum albumin, slides were incubated at 4 °C overnight with primary antibodies. After 3 times of PBS-T wash, secondary antibodies were incubated for 1 h, RT. For immune-reactive TUNEL, reagents were treated at 37 °C for 1 h. After 3 times of PBS-T wash, slides were mounted in ProLong Gold anti-fade DAPI reagent (1,674,645, Life technologies) and examined using a microscope (DMi8, Leica) in dark area. Primary antibodies used were: Ki67 (GeneTex, GTX16667) and TUNEL (Roche, 11,684,795,910 In Situ Cell Death Detection Kit). Secondary antibodies used were: Anti-rabbit (A21207, Life technologies).

### RNA isolation, reverse transcription and qRT-PCR

Total RNA extracts from mouse liver, Hep3B cells, and Raw 264.7 cells were prepared using the TRIzol® Reagent (Thermo Fisher Scientific, MA, USA), Chloroform (C2432, Sigma), Isopropanol (1.09634.1011, Merck), and DEPC (E174, Amresco). Reverse transcription was performed with 1.5 μg of total RNA and Reverse transcriptase kit (SG-cDNAS100, Smartgene, United Kingdom) following manufacturer’s protocol. Quantitative PCR(real-time PCR) was carried out using specific primers (Table 1), Excel Taq Q-PCR Master Mix (SG-SYBR-500, Smartgene) and Stratagene Mx3000P(Agilent Technologies) equipped with a 96-well optical reaction plate. Negative controls, containing water instead of sample cDNA, were used in each plate. All experiments were run in triplicate and mRNA values were calculated based on the cycle threshold and monitored for a melting curve.

### Mitochondrial stress test

Raw 264.7 cells were grown in DMEM, 10% FBS, and 1% penicillin/streptomycin media and incubated with steroid starvation media (w/o phenol red, 2% CD-FBS, 1% penicillin/streptomycin) for 24 h. After removing endogenous steroid hormones, genistein (1 μM) was treated for 18 h. To remove the carbon sources and buffering agents from the cell media, cells were incubated in non-sodium bicarbonate media in non-CO_2_ incubator in 37 °C, 1 h. Cells were then proceeded to Seahorse XFp Extracellular Flux Analyzer (Agilent, Korea). Oligomycin (2 μM), FCCP (0.5 μM), and rotenone/antimycin A (0.5 μM) were used for analyze. Number of samples used for experiment was 3 for each group.

### Statistical analysis

Data are reported as mean ± SEM. Differences between means were obtained by Student’s t-test and the one-way ANOVA followed by a Dunnett post analysis was performed using Graph Pad Software (GraphPad Inc., San Diego, CA).

## Results

### Genistein suppresses the development of DEN-induced HCC in menopausal mice

**Fig. 1:**
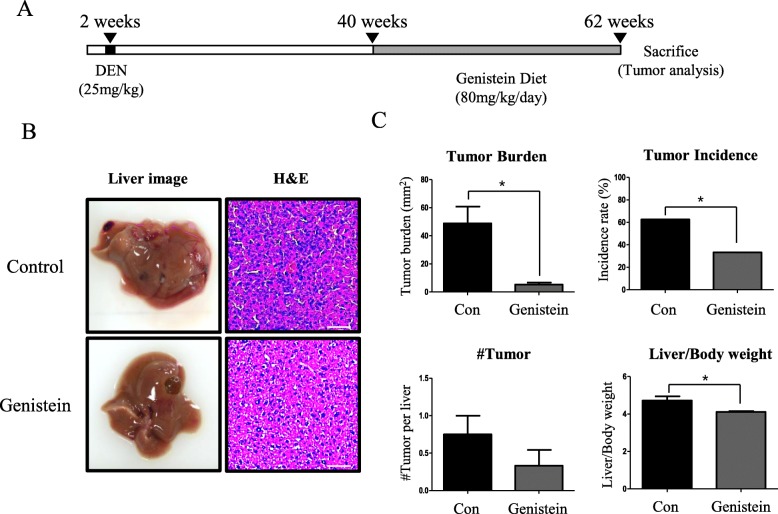
Dietary intake of genistein delays initiation and suppresses development of HCC. **a **Schedule of the dietary intake of genistein. HCC bearing mice were fed genistein with combined chow for 5 months from the age of 40 weeks to 62 weeks. **b **Representative images showing HCC development in 62-week-old livers of female control and genistein groups that were DEN-treated mice. Hematoxylin and eosin (H&E) staining (Scale bar, 20 μm) of liver is presented. Pyknotic nuclei in tumor cell is stained with dark hematoxylin. **c **Tumor burden (mm^2^) quantified by Image J, incidence, numbers, and liver weight per body weight in 62-week-old livers of female control and genistein groups. Values represent means ± SEM. *, *p* < 0.05. Numbers of mice used in experiment is at least each 6 for control and genistein groups

To develop artificial HCC, diethylnitrosamine (DEN) was injected to 2-week-old female C57BL/6 mice. At the age of 40 weeks, some mice were randomly fed genistein (Fig. 1a). After 5 months of feeding, the dietary intake of genistein remarkably reduced the incidence of HCC in 62-week-old mice. Representative images of livers from control and genistein groups are shown in Fig. 1b. Pyknotic nuclei observed in aggregated tumor cells were easily recognized by dark hematoxylin staining (Fig. 1b). The tumors presented white-to-yellow nodules, and the ratio of tumor burden per liver significantly decreased in the genistein group (13.2%, *p* < 0.05), relative to the control group (Fig. 1c). The HCC incidence rate significantly reduced in the genistein group (53.3%, *p* < 0.05), compared to the control group, although there was not a significant decrease in tumor number (Fig. 1c). As liver weight increases due to HCC, the ratio of liver weight per body weight also significantly decreased in the genistein group (88.8%, *p* < 0.05), compared to the control group (Fig. 1c).

**Table 1:** Primers used for real-time or conventional PCR, related to Figs. 3 and 5.

Gene name	Upper primer (5′ - 3′)	Lower primer (5′ - 3′)	Species
*IκBα*	TGA AGG ACG AGG AGT ACG AGC	TTC GTG GAT GAT TGC CAA GTG	Mouse
*IL-6*	CTG CAA GAG ACT TCC ATC CAG	AGT GGT ATA GAC AGG TCT GTT GG	Mouse
*TNF*	CCT GTA GCC CAC GTC GTA G	GGG AGT AGA CAA GGT ACA ACC C	Mouse
*Aifm1*	TCC AGA GGC CGA AAC AGA G	CAT TTT GCC CCC TGA TGA ACC	Mouse
*Ndufb5*	CGA GCT TGC AGA AAT CCC AGA AGG C	GTC CAT CAC CTC GGG CAC GCA TCA G	Mouse
*Rplp0*	GCA GCA GAT CCG CAT GTC GCT CCG	GAG CTG GCA CAG TGA CCT CAC ACG G	Mouse
*Bcl2*	ATG CCT TTG TGG AAC TAT ATG GC	GGT ATG CAC CCA GAG TGA TGC	Mouse
*Bax*	TGA AGA CAG GGG CCT TTT TG	AAT TCG CCG GAG ACA CTC	Mouse
*Mdm2*	TGT CTG TGT CTA CCG AGG GTG	TCC AAC GGA CTT TAA CAA CTT CA	Mouse

### Genistein increases phospho-AMPK in whole liver, hepatocytes, and macrophages

**Fig. 2:**
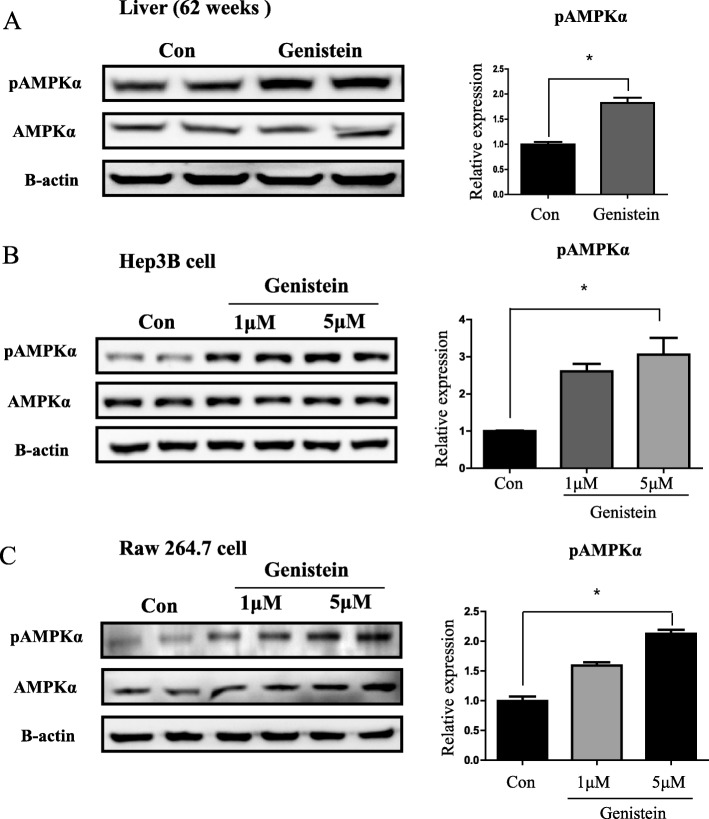
Increased levels of phospho-AMPKα in liver of 62-week-old mice, Hep3B cell, and Raw 264.7 cell. **a **Western blot analysis and quantification of phosphor-AMPKα in liver of 62-week-old mice. Beta actin was used as an internal control. **b** Western blot analysis and quantification of phosphor-AMPKα in Hep3B cell line. Beta actin was used as an internal control. Genistein was treated for 6 h after steoid starvation with charcoal dextran fetal bovine serum (CD-FBS) for overnight. **c** Western blot analysis and quantification of phospho-AMPKα in Raw 264.7 cell. Beta actin was used as an internal control. Genistein was treated for 6 h after steroid starvation with CD-FBS for overnight. Values represent means ± SEM of at least 3 experiments. *, *P* < 0.05. Numbers of mice used in experiment is at least each 6 for control and genistein groups

As an important mediator for inflammation and apoptosis, we first analyzed AMPK levels, especially phospho-AMPK, in genistein treated mice. In the livers of 62-week-old mice, we observed significant increases (1.82 fold, *p* < 0.05) of phospho-AMPKα in the genistein group, compared to the control group (Fig. 2a). Likewise, we treated a Hep3B cell line with genistein for 6 h and observed significantly (*p* < 0.05) increased levels of phospho-AMPKα at 1 μM (2.61 fold) and 5 μM (3.06 fold) genistein, compared to the control group (Fig. 2b). Moreover, in mouse macrophages (Raw 264.7 cell), phospho-AMPKα levels significantly increased (2.13 fold, *p* < 0.05) in the genistein treated group (5 μM), compared to the control group (Fig. 2c). Through these results, we confirmed that genistein activates AMPKα to its phospho-form.

### Genistein induces apoptosis through an AMPK-mediated caspase pathway

**Fig. 3:**
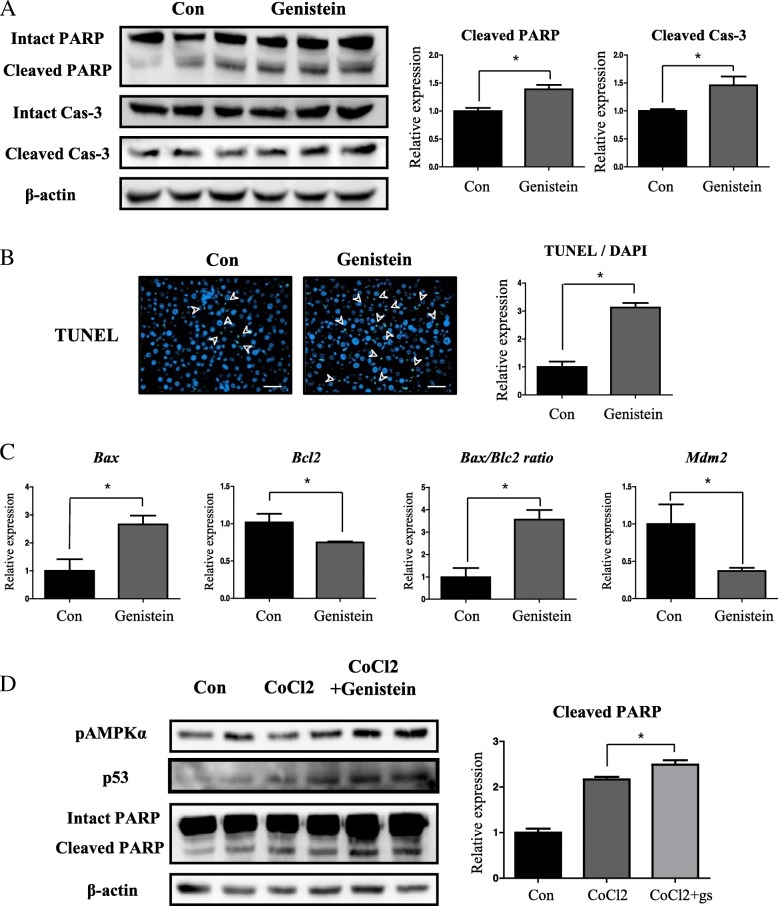
Genistein induces apoptosis through AMPK activation involving caspase pathways. **a** Western blot analysis and quantification intact PARP, cleaved PARP, intact Cas-3, and cleaved Cas-3 in livers of 62-week-old mice. Beta actin was used for an internal control. **b** Immunofluorescence detection of TUNEL (green) as a marker of apoptosis in livers of control and genistein groups. Nuclei are stained with DAPI (blue). White arrowheads indicate apoptotic cells (Scale bar, 50 μm). (**c**) qRT-PCR analysis of apoptotic genes involved in p53 pathway. **d** Western blot analysis of phosphor-AMPKα, p53, intact PARP, and cleaved PARP. Western blot quantification of cleaved PARP in Hep3B cell after treatment of CoCl2 (100 μM) and genistein (1 μM). Beta actin was used as an internal control. Values represent means ± SEM of at least 3 experiments. *, *P* < 0.05

To investigate whether apoptosis was induced by genistein anti-cancer activity, we first analyzed levels of apoptotic markers in the livers of 62-weeks-old mice. Cleaved PARP showed significantly increased levels (1.39 fold, *p* < 0.05) in the livers of the 62-week genistein group, relative to the control group (Fig. 3a). Cleaved Cas-3 also showed significantly increased levels (1.4 fold, p < 0.05) in the livers of the 62-week genistein group, compared to the control group (Fig. 3a). Moreover, the number of terminal deoxynucleotidyl transferase dUTP nick end labeling (TUNEL) positive signals, which is indicative of apoptosis, increased (3.12 fold, *p* < 0.05) in the genistein group, relative to the control group, when the TUNEL signals were divided by the number of positive signals for 4′, 6-diamidino-2-phenylindole (DAPI), which is a dye that labels the nucleus (Fig. 3b). The mRNA level of *Bax* increased (2.65 fold) and that of *Bcl2* decreased (73.2%) in the genistein group (*p* < 0.05, Fig. 3c). The ratio between Bax and Bcl2, which an apoptosis marker, significantly increased (3.62 fold, *p* < 0.05) in the genistein group, relative to the control group (Fig. 3c). Furthermore, the level of Mdm2, a p53 transcriptional repressor, also significantly decreased (37%, *p* < 0.05) in the genistein group, compared to the control group (Fig. 3c).

Since apoptosis is important for hepatocytes, we made use of a Hep3B in vitro system to investigate effects of genistein on hepatocyte apoptosis. While the level of phospho-AMPKα increased (1.89 fold, *p* < 0.05) upon CoCl2 + genistein, relative to the than CoCl2 group, the level of p53 also increased (1.4 fold, *p* < 0.05) concomitantly in the CoCl2 + genistein group (Additional file 1: Figure S1). Accordingly, the level of cleaved PARP, an executioner of apoptosis, significantly increased (2.16 fold, *p* < 0.05) in the CoCl2 group, relative to the control group (Fig. 3d), and further increased (1.15 fold, *p* < 0.05) in the CoCl2 + genistein group, relative to the CoCl2 group (Fig. 3d).

### Genistein suppresses hepatic DEN-induced inflammation and tumor proliferation

**Fig. 4:**
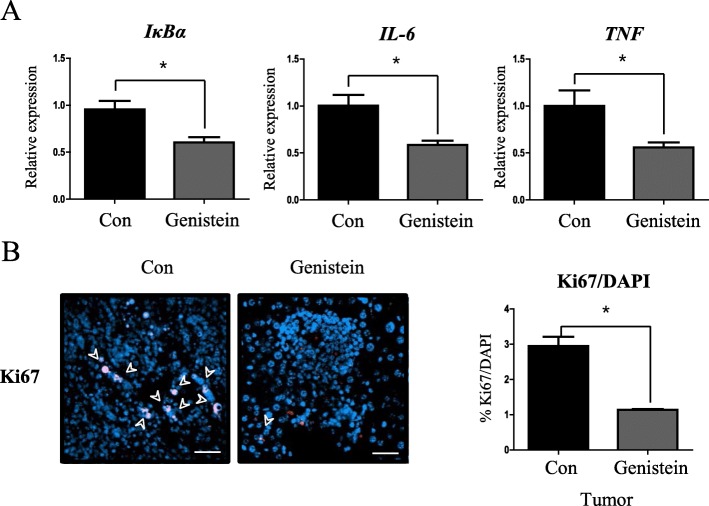
Genistein suppressed hepatic inflammation and tumor proliferation. (**a**) qRT-PCR analysis of IκBα and pro-inflammatory cytokines in liver of 62-week-old mice. *Rplp0* was used for an internal control. (**b**) Ki67 positive cells (pink) are shown. Nuclei are stained with DAPI (blue). White arrowheads indicate proliferating cells (Scale bar, 50 μm). Ki67 per DAPI (%) in tumor area of control and genistein groups. Values represent means ± SEM of at least 3 experiments. *, *P* < 0.05

Next, we analyzed the level of the inflammatory markers that are considered as typical accelerants for HCC initiation. We found that the genistein group showed significantly decreased (76.5%, *p* < 0.05) *IκBα* mRNA levels than the control group (Fig. 4a), which suggests that NF-κB, a key player in pro-inflammation, is suppressed. The mRNA levels of pro-inflammatory cytokines, IL-6 (68%) and TNF (76.4%), both significantly (*p* < 0.05) decreased in the genistein group, compared to the control group (Fig. 4a). As inflammation triggers tumor proliferation, we used Ki67 as a proliferation marker and observed significant decrease (38.6%, *p* < 0.05) in the genistein group, compared to the control group, when Ki67 signal was divided by the number of DAPI-positive signals (Fig. 4b).

To evaluate the effects of genistein in acute hepatic injury, we treated mice with DEN (100 mg/kg) for 48 h, after injecting genistein (200 mg/kg) for 24 h. Ovariectomies were performed to terminate endogenous estrogen, and a subsequent restoration was maintained for 2 weeks. In the microscopic analysis of H&E staining, the DEN group showed markedly damaged regions, while the DEN-genistein group exhibited less damaged areas than the DEN group (Additional file 1: Figure S2A). This result closely associated with decreased (79.1%, *p* < 0.05) alanine aminotransferase (ALT) levels in the DEN-genistein group than in the DEN group, and the disruption of hepatocytes by DEN injection increased the level of ALT (1.63 fold, *p* < 0.05), which is a liver cell damage marker (Additional file 1: Figure S2B). Furthermore, the mRNA level of *IκBα* increased (4.77 fold, *p* < 0.05) in DEN the group, compared to the control group, which implies that the inflammation was triggered by DEN (Additional file 1: Figure S2B). In contrast, decreased (47.7%, *p* < 0.05) *IκBα* mRNA levels were observed in the DEN-genistein group, compared to the DEN group, which implies that the inflammatory effect was suppressed by genistein (Additional file 1: Figure S2B).

### Genistein promotes mitochondrial respiration in macrophages through AMPK activation

**Fig. 5:**
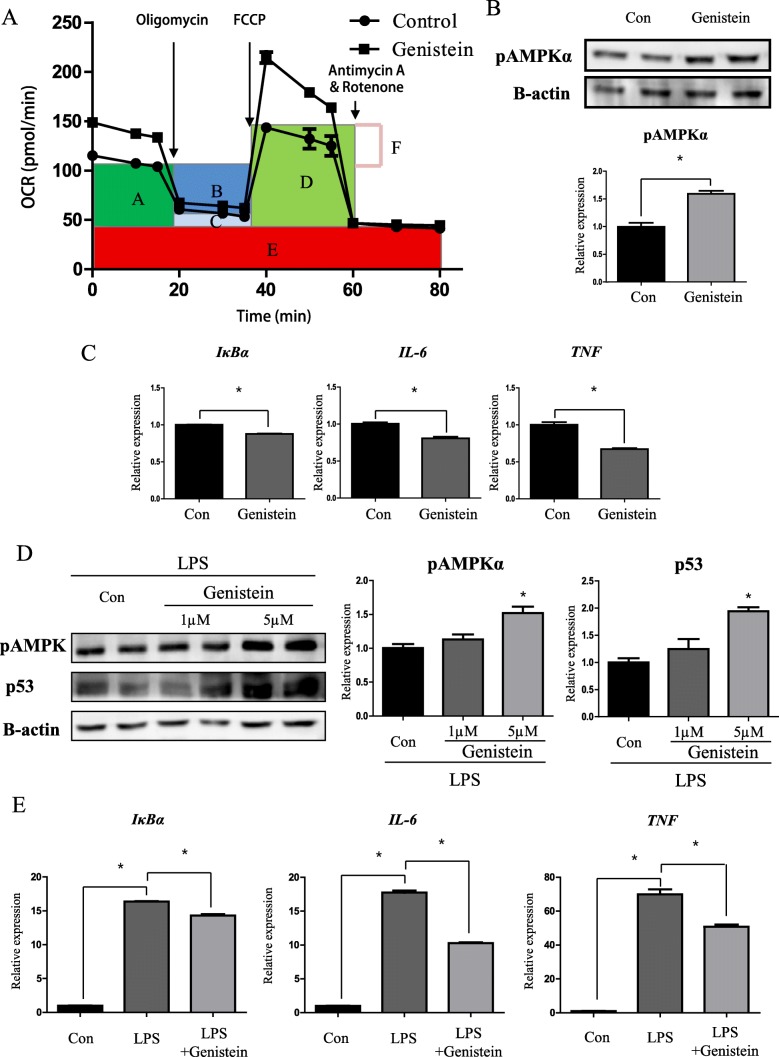
Genistein induced mitochondrial respiration and suppressed pro-inflammatory state in macrophage. **a** Mitochondrial respiration analysis of control and genistein groups (1 μM, 18 h). A: Basal respiration, B: ATP production, C: Proton Leak, D: Maximal respiration, E: Non-mitochondrial respiration, F: Spatial respiratory capacity. **b** Western blot analysis and quantification of phospho-AMPKα. Genistein (1 μM) was treated for 6 h. Beta actin was used as an internal control. (**c**) qRT-PCR analysis of pro-inflammatory cytokines. Genistein (1 μM) was treated for 6 h. *Rplp0* was used as an internal control. **d** Western blot analysis and quantification of phospho-AMPKα and p53. LPS (100 ng/ml) was treated for 3 h and genistein (1 μM) was pre-treated for 2 h. Beta actin was used as an internal control. (**e**) qRT-PCR analysis of IκBα and pro-inflammatory cytokines in Raw 264.7 cell. *Rplp0* was used for an internal control. Genistein (1 μM) was pre-treated 1 h before LPS incubation (100 ng/ml, 3 h). Values represent means ± SEM of at least 3 experiments. *, *P* < 0.05

As pro-inflammatory cytokine levels were decreased by genistein, we hypothesized that genistein triggers an anti-inflammatory macrophage state of by inducing mitochondrial respiration through AMPK activation. To investigate how genistein influences macrophage mitochondrial respiration, we evaluated the metabolic status (Oxygen consumption rate, OCR) of Raw 264.7 cells after genistein treatment (1 μM) for 18 h. As shown in Fig. 5a, genistein treatment triggered the overall induction of mitochondrial respiration in Raw 264.7 cells. First, the basal respiration level significantly increased in the genistein group (1.45 fold, *p* < 0.05), compared to the control group (Additional file 1: Figure S3A-A). When we blocked ATP synthase with oligomycin, we observed increased ATP production levels (1.45 fold, *p* < 0.05) in the genistein group, compared to the control group (Additional file 1: Figure S3A-B). Moreover, increased proton leak levels were observed in the genistein group (1.44 fold), relative to the control group, which indicated the uncoupling of non-mitochondrial respiration, similar to the effects observed with rotenone/antimycin A (Additional file 1: Figure S3A-C, E). In the presence of p-trifluoromethoxyphenylhydrazone (FCCP), which blocks mitochondrial respiration, a huge rise in oxygen consumption rate was observed in the genistein group, relative to the control group, which suggests that maximal respiration (1.73 fold) and spare respiratory capacity (2.32 fold) considerably increase after genistein treatment (*p* < 0.05, Additional file 1: Figure S3A-D, F).

The induction of oxidative phosphorylation consequently led to a reduced pro-inflammatory state of Raw 264.7 cells. After 6 h of genistein (1 μM) incubation, which was similar to lipopolysaccharide (LPS) conditioning, we observed significantly (*p* < 0.05) suppressed levels of *IκBα* (87%), *IL-6* (81%) and *TNF* (67%) in the genistein group, compared to the control group (Fig. 5c). This observation concomitantly occurred with the induction of phospho-AMPK by genistein (Fig. 5b). Similarly, the induction of phospho-AMPK was also observed in the LPS-triggered inflammatory state. After pre-incubation with genistein for 2 h, Raw 264.7 cells were exposed to LPS (100 ng/ml) for 3 h, after which the phospho-AMPK levels significantly increased (1.52 fold, *p* < 0.05) in the LPS-genistein group (5 μM), compared to the LPS-control group (Fig. 5d).

phospho-AMPK may associate with mitochondrial respiration through p53, as we observed significantly increased (1.94 fold, *p* < 0.05) levels of p53 in the LPS-genistein group (5 μM), relative to the LPS-control group (Fig. 5e). Following the induction of p53, its downstream target, *Aifm1*, also showed significantly (*p* < 0.05) increased (1.74 and 1.55 fold) levels after genistein treatment (1 μM and 5 μM), compared to the LPS group (Additional file 1: Figure S4). This result is consistent with the induction of mitochondrial respiration, as Aif promotes oxidative phosphorylation. As a result, LPS triggered acute inflammation in Raw 264.7 cells and increased (16.37 fold) *IκBα* mRNA levels, relative to the control group, while genistein suppressed (87.5%) inflammation in the LPS group (p < 0.05, Fig. 5e). Moreover, the mRNA levels of IL-6 (57.9%) and TNF (73%) decreased in the LPS + genistein group, compared to the LPS group. These mRNA levels were also induced in the LPS group (17.8 fold and 69.3 fold), relative to the control group (*p* < 0.05, Fig. 5e).

## Discussion

While HCC occurs predominantly in patients with underlying chronic liver disease and cirrhosis, genistein consumption has a high potential to prevent the progression of severe liver diseases into liver cancer [7]. Numerous studies have investigated genistein and liver cancer cells in vitro [21, 22]; however, the long-term effects of genistein on HCC establishment and development in vivo remain unclear [23]. Therefore, our study highlights the effects of dietary genistein intake on long-standing HCC suppression. We found that genistein suppresses cancer initiation and development through AMPK-mediated anti-inflammation and pro-apoptosis.

Inflammatory and apoptotic activities in macrophages and hepatocytes are triggered by characterized signals during HCC development. In the present study, genistein consistently increased phospho-AMPK levels in liver, Hep3B cell, and Raw 264.7 cells. AMPK is known to suppress inflammation, which usually implies poor prognosis and development of HCC [24], through reducing pro-inflammatory marker and NF-kB levels [25]. NF-kB is the main contributor to hepatocarcinogenesis with chronic inflammation and functions through inducing the levels of growth factors and compensatory regeneration [26]. Subsequent to NF-kB stimulation, the release of inflammatory cytokines leads to the proliferation and survival of cancer cells, which promotes cancer development [27]. In the present study, genistein down-regulated TNF and IL-6, among the pro-inflammatory cytokines secreted by NF-kB signaling. TNF is implicated with cellular proliferation in hepatocarcinogenesis and persistent IL-6 is associated with tumor development [28, 29]. Through our result of Ki67 immunofluorescence analysis, which is a marker for vigorous cancer cell mitosis, we observed that genistein consequently suppressed the proliferation of cancer cells [30].

Importantly, genistein promoted anti-cancer apoptotic activity by inducing phospho-AMPK. Contrary to inflammation, apoptosis is a programmed cell death pathway that suppresses cancer activity [31]. Therefore, the down-regulation of anti-apoptotic factors through HCC therapy can suppress the growth of cancer cells [32]. As a tumor suppressor gene that reacts with stress responses and contains anti-proliferative activity, p53 regulates the transcription of apoptotic effector genes and consequently induces caspases to promote apoptosis [33]. AMPK is also involved with p53 in apoptosis [15], and we observed p53 induction and AMPK phosphorylation by genistein. Moreover, we observed that genistein increased the level of *Bax* and decreased the level of *Bcl2* in the present study, which are known to be involved in the p53 pathway [34]. The decreased level of *Mdm2* was also consistent with the increased level of p53, as Mdm2 is a repressor of p53-mediated apoptosis [35]. More importantly, the increased level of p53 by genistein consequently led to the induction of apoptotic markers. As an executioner for apoptosis [36], the level of cleaved Cas-3 and cleaved PARP increased upon genistein treatment. The increased number of positive TUNEL signals upon genistein treatment also demonstrates the promotion of apoptosis [37]. Interestingly, we found that the apoptotic effect of genistein involves AMPK phosphorylation, and a recent study found that genistein has a synergistic effect with sorafenib, which induces apoptosis in HCC cells [38].

Furthermore, we observed that genistein increased mitochondrial function in Raw 264.7 cells through the induction of phospho-AMPK and p53. AMPK is known to induce mitochondrial respiration, and p53 also ensures mitochondrial function through Aif-1 induction, by sensitizing oxidative phosphorylation when apparent apoptotic signs are minimal [39]. In our seahorse data, we observed increased mitochondrial ATP production and *Aif-1* levels upon the induction of p53. Considering that increased oxidative phosphorylation attenuates pro-inflammatory macrophage responses, and mitochondrial dysfunction prevents repolarization to anti-inflammatory M2 macrophages [40–42], these results are consistent with the down-regulation of pro-inflammatory cytokines in Raw 264.7 cells and suggests that genistein induces the anti-inflammatory state of macrophages. In addition, the increased p53 levels in Raw 264.7 cells upon genistein treatment implies that AMPK is consistently involved in oxidative phosphorylation, as AMPK induces p53 and promotes macrophage polarization to an anti-inflammatory state [42]. For cancer therapy, mitochondrial oxidative phosphorylation increases the sensitivity of HCC to drugs [43], and the decrease in glycolysis leads to HCC suppression [44]. A previous study has suggested that genistein reinforces cisplatin activity [45], and our study also suggests the potential of genistein to improve or sensitize the action of HCC drugs.

According to the present study, the long-term dietary intake of genistein remarkably suppresses hepatocarcinogenesis and hepatocellular carcinoma development through AMPK activation. Phosphorylated AMPK plays potential roles in anti-inflammation, pro-apoptotic, and pro-oxidative phosphorylation process in HCC. While chemotherapy still exerts severe damage to the host, the dietary intake of genistein is highly valuable for HCC prevention, when intake is consistent and maintained for long time, since genistein does not have an immediate effect.

## Conclusions

Our study reveals the long-term effects of dietary genistein intake, which prevents the initiation and suppresses the development of HCC, through mechanisms involving AMPK.

## Additional file

Additional file 1: **Figure S1.** Western blot quantification of pAMPKα and p53 in Hep3B cell. CoCl2+genistein group showed significant increase (1.89 fold and 1.4 fold, *p*<0.05) than CoCl2 group in mRNA level of pAMPKα and p53. Beta actin was used for an internal control. CoCl2 (100μM) and genistein (1μM) were treated for 48 hrs after steroid starvation for overnight. Values represent means ± SEM of at least 3 experiments. *, *P*<0.05. **Figure S2.** Genistein ameliorated acute hepatic injury by DEN treatment. DEN was treated (100mg/kg) for 48 hrs after injecting genistein (200mg/kg) for 24 hrs previously. Ovariectomy was performed to terminate endogenous estrogen, and subsequent restoration was maintained for 2 weeks. (A) Hematoxylin & eosin staining (Scale bar, 200μm) of liver was presented. DEN showed remarkarbly increased damage area while genistein suppressed it. (B) qRT-PCR analysis of *IκBα* is presented. DEN group showed significant increase (4.77 fold, *p*<0.05)in mRNA level of *IκBα* than vehicle group. DEN-genistein grouop showed significant decrease (47.7%, *p*<0.05) in mRNA level of *IκBα* than DEN group. *Rplp0* was used for an internal control. As a hepatic damage marker, ALT level (U/I) showed significant increase (1.63 fold, p<0.05) in DEN group than vehicle group. DEN-genistein grouop showed significant decrease (79.1%, p<0.05) in ALT level than DEN group. Values represent means ± SEM of at least 3 experiments. *, *P*<0.05. **Figure S3.** Overall increased levels of oxygen consumption rate (OCR, pmol/min) for each steps during mitochondrial stress test. Genistein (1μM) was treated for 18 hrs after steroid starvation for overnight. Values represent means ± SEM of at least 3 experiments. *, *P*<0.05. **Figure S4.** Relative mRNA level of apoptotic gene involved in p53 pathway and oxidative phosphorylation in Raw 264.7 cell. qRT-PCR analysis of *Aifm1* is presented. *Rplp0* was used for an internal control. Genistein (1μM , 5μM) was treated for 6 hrs after steroid starvation for overnight. Values represent means ± SEM of at least 3 experiments. *, *P*<0.05. (DOCX 277 kb)
