# Supplementary material for: Dietary intake of genistein suppresses hepatocellular carcinoma through AMPK-mediated apoptosis and anti-inflammation
Source: BMC Cancer. 2019 Jan 3;19:6. doi: 10.1186/s12885-018-5222-8 (PMC6318960; doi:10.1186/s12885-018-5222-8)
Supplement: Supplementary file 1 — Figure S1. Western blot quantification of pAMPKα and p53 in Hep3B cell. CoCl2+genistein group showed significant increase (1.89 fold and 1.4 fold, p<0.05) than CoCl2 group in mRNA level of pAMPKα and p53. Beta actin was used for an internal control. CoCl2 (100μM) and genistein (1μM) were treated for 48 hrs after steroid starvation for overnight. Values represent means ± SEM of at least 3 experiments. *, P<0.05. Figure S2. Genistein ameliorated acute hepatic injury by DEN treatment. DEN was treated (100mg/kg) for 48 hrs after injecting genistein (200mg/kg) for 24 hrs previously. Ovariectomy was performed to terminate endogenous estrogen, and subsequent restoration was maintained for 2 weeks. (A) Hematoxylin & eosin staining (Scale bar, 200μm) of liver was presented. DEN showed remarkarbly increased damage area while genistein suppressed it. (B) qRT-PCR analysis of IκBα is presented. DEN group showed significant increase (4.77 fold, p<0.05)in mRNA level of IκBα than vehicle group. DEN-genistein grouop showed significant decrease (47.7%, p<0.05) in mRNA level of IκBα than DEN group. Rplp0 was used for an internal control. As a hepatic damage marker, ALT level (U/I) showed significant increase (1.63 fold, p<0.05) in DEN group than vehicle group. DEN-genistein grouop showed significant decrease (79.1%, p<0.05) in ALT level than DEN group. Values represent means ± SEM of at least 3 experiments. *, P<0.05. Figure S3. Overall increased levels of oxygen consumption rate (OCR, pmol/min) for each steps during mitochondrial stress test. Genistein (1μM) was treated for 18 hrs after steroid starvation for overnight. Values represent means ± SEM of at least 3 experiments. *, P<0.05. Figure S4. Relative mRNA level of apoptotic gene involved in p53 pathway and oxidative phosphorylation in Raw 264.7 cell. qRT-PCR analysis of Aifm1 is presented. Rplp0 was used for an internal control. Genistein (1μM , 5μM) was treated for 6 hrs after steroid starvation for overnight. Values represent [file 12885_2018_5222_MOESM1_ESM.docx]

**Supplementary data**

**Dietary intake of genistein suppresses hepatocellular carcinoma through AMPK mediated anti-inflammation and pro-apoptosis.**

Sang R. Lee, Sun Woo Kwon, Young Ho Lee, Pelin Kaya, Jong Min Kim, Changhwan Ahn, Eui-Man Jung, Geun-Shik Lee, Beum-Soo An, Eui-Bae Jeung, Bae-keun Park, and Eui-Ju Hong

**Table contents**

Supplementary Fig. 1 ……………………………………………………………..………...2

Supplementary Fig. 2 …………………………………………………………..…………...3

Supplementary Fig. 3 …………………………………………………………..…………...4

Supplementary Fig. 4 …………………………………………………………..…………...4


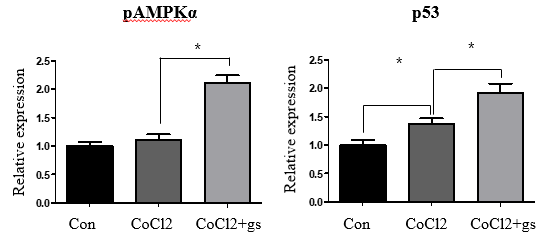


Figure S1. Western blot quantification of pAMPKα and p53 in Hep3B cell. CoCl2+genistein group showed significant increase (1.89 fold and 1.4 fold, p<0.05) than CoCl2 group in mRNA level of pAMPKα and p53. Beta actin was used for an internal control. CoCl2 (100µM) and genistein (1µM) were treated for 48 hrs after steroid starvation for overnight. Values represent means ± SEM of at least 3 experiments. *, P<0.05.


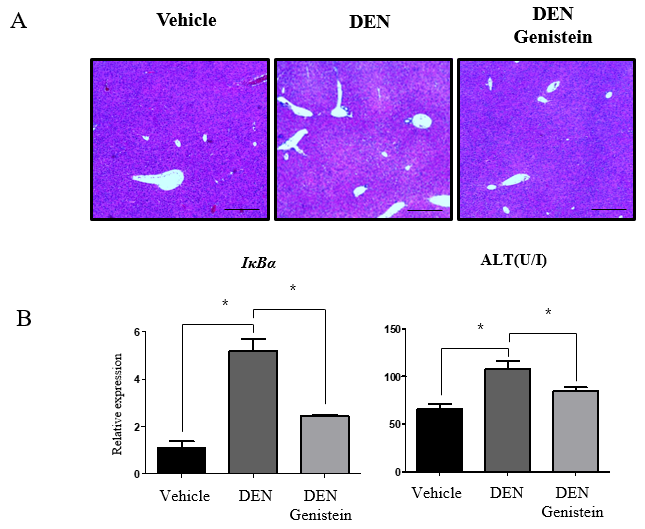


Figure S2. Genistein ameliorated acute hepatic injury by DEN treatment. DEN was treated (100mg/kg) for 48 hrs after injecting genistein (200mg/kg) for 24 hrs previously. Ovariectomy was performed to terminate endogenous estrogen, and subsequent restoration was maintained for 2 weeks. (A) Hematoxylin & eosin staining (Scale bar, 200µm) of liver was presented. DEN showed remarkarbly increased damage area while genistein suppressed it. (B) qRT-PCR analysis of *IκBα* is presented. DEN group showed significant increase (4.77 fold, p<0.05)in mRNA level of *IκBα* than vehicle group. DEN-genistein grouop showed significant decrease (47.7%, p<0.05) in mRNA level of *IκBα* than DEN group. *Rplp0* was used for an internal control. As a hepatic damage marker, ALT level (U/I) showed significant increase (1.63 fold, p<0.05) in DEN group than vehicle group. DEN-genistein grouop showed significant decrease (79.1%, p<0.05) in ALT level than DEN group. Values represent means ± SEM of at least 3 experiments. *, P<0.05.


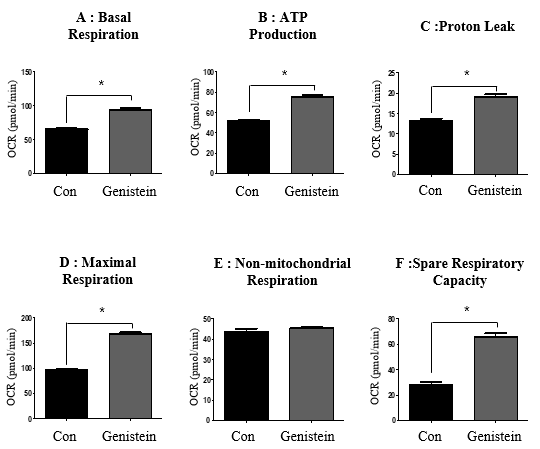


Figure S3. Overall increased levels of oxygen consumption rate (OCR, pmol/min) for each steps during mitochondrial stress test. Genistein (1µM) was treated for 18 hrs after steroid starvation for overnight. Values represent means ± SEM of at least 3 experiments. *, P<0.05.


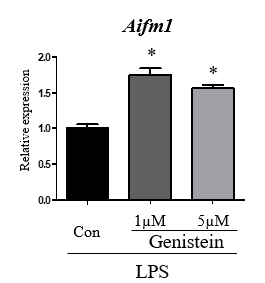


Figure S4. Relative mRNA level of apoptotic gene involved in p53 pathway and oxidative phosphorylation in Raw 264.7 cell. qRT-PCR analysis of *Aifm1* is presented. *Rplp0* was used for an internal control. Genistein (1µM , 5µM) was treated for 6 hrs after steroid starvation for overnight. Values represent means ± SEM of at least 3 experiments. *, P<0.05.
